# Identification of miRNAs and genes for predicting Barrett’s esophagus progressing to esophageal adenocarcinoma using miRNA-mRNA integrated analysis

**DOI:** 10.1371/journal.pone.0260353

**Published:** 2021-11-24

**Authors:** Chengjiao Yao, Yilin Li, Lihong Luo, Qin Xiong, Xiaowu Zhong, Fengjiao Xie, Peimin Feng

**Affiliations:** 1 Hospital of Chengdu University of Traditional Chinese Medicine, Chengdu, Sichuan, China; 2 Department of Geriatrics of the Affiliated Hospital, North Sichuan Medical College, Nanchong, Sichuan, China; 3 Department of Laboratory Medicine, North Sichuan Medical College, Nanchong, Sichuan, China; 4 Department of Clinical Laboratory, Affiliated Hospital of North Sichuan Medical College, Nanchong, Sichuan, China; Institute of Parasitology and Biomedicine, SPAIN

## Abstract

Barrett’s esophagus (BE) is defined as any metaplastic columnar epithelium in the distal esophagus, which predisposes to esophageal adenocarcinoma (EAC). Yet, the mechanism through which BE develops to EAC still remain unclear. Moreover, the miRNA-mRNA regulatory network in distinguishing BE from EAC still remains poorly understood. To identify differentially expressed miRNAs (DEMs) and genes (DEGs) between EAC and BE from tissue samples, gene expression microarray datasets GSE13898, GSE26886, GSE1420 and miRNA microarray datasets GSE16456, GSE20099 were downloaded from Gene Expression Omnibus (GEO) database. GEO2R was used to screen the DEMs and DEGs. Pathway and functional enrichment analysis were performed by DAVID database. The protein–protein interaction (PPI) network was constructed by STRING and been visualized by Cytoscape software. Finnal, survival analysis was performed basing TCGA database. A total of 21 DEMs were identified. The enriched functions and pathways analysis inclued Epstein-Barr virus infection, herpesvirus infection and TRP channels. GART, TNFSF11, GTSE1, NEK2, ICAM1, PSMD12, CTNNB1, CDH1, PSEN1, IL1B, CTNND1, JAG1, CDH17, ITCH, CALM1 and ITGA6 were considered as the hub-genes. Hsa-miR-143 and hsa-miR-133b were the highest connectivity target gene. JAG1 was predicted as the largest number of target miRNAs. The expression of hsa-miR-181d, hsa-miR-185, hsa-miR-15b, hsa-miR-214 and hsa-miR-496 was significantly different between normal tissue and EAC. CDH1, GART, GTSE1, NEK2 and hsa-miR-496, hsa-miR-214, hsa-miR-15b were found to be correlated with survival.

## 1. Introduction

Esophageal carcinoma (EC) is the eighth most common cancer in the world. A total of 17650 new cases and 16080 deaths have been reported in 2019 [1]. The mortality rate is significantly higher in males than in females, and the overall five-year survival rate is only 19% [1]. EC is usually classified into esophageal squamous cell carcinoma (ESCC) and esophageal adenocarcinoma (EAC). There are several accepted hypotheses concerning which cells give rise to EAC in adults. The most plausible one is that EAC develops according to the following process: normal esophageal epithelium → hyperplasia of proper esophageal gland → dentate line Migration → Barrett’s esophagus (BE) → EAC [2]. From the conversional process, BE is the only recognized precursor of EAC. Patients with BE are almost 30–120 times more likely to develop EAC [3]. However, the mechanism through which BE develops to EAC and relevant driving factors still remain unclear. Therefore, the identification of key molecular biomarkers for predicting BE, implementing the strategy of clinical risk stratification, and focusing on the higher risk patient may be critical in preventing EAC.

Over recent years, a number of studies examined specific patterns of gene transcript levels in EAC. So far, many significant genes have been associated with the pathogenesis of EAC. For example, a tumor suppressor gene TP53 is one of the first genes that was examined in Barrett’s-associated neoplasms. Studies have found that patients with loss of TP53 are almost 16 times more likely to develop EAC compared to those with normal expression of TP53 [4]. Moreover, a decreased expression of p14ARF has been suggested as a biomarker for disease progression, from normal epithelium to non-dysplastic BE and even to EAC [4]. MMP1 gene, which participates in numerous inflammatory processes of cancer, has shown to be up-regulated in EAC and BE samples [5]. COL1A1 has shown to be a potential biomarker for distinguishing EAC from BE [3].

MicroRNAs (miRNAs) are a group of small non-coding RNA molecules that contain approxinately 18 to 25 nucleotides. It has been described that miRMAs participate in a series of biological processes as a post-transcriptional regulators. Aberrant expression of miRNAs has been associated with the development of BE. For instance, miR-215, which acts as a tumor suppressor by promoting apoptosis, is low in the normal squamous epithelium and high in BE [6]. In BE, miR-196a which targets KRT5 and SPRR2C, has been suggested to be a potential biomarker for the disease progression into EAC [7]. Still, the miRNA-mRNA regulatory network remains poorly understood in distinguishing BE from EAC.

In this research, we identified differentially expressed genes (DEGs) and differentially expressed miRNAs (DEMs) between EAC and BE from biopsies. The aim of this study was to seek possible potential biomarkers and molecular mechanisms for clinical risk stratification strategies for EAC.

## 2. Materials and methods

### 2.1. Microarray data collection

First, “Barrett’s esophagus” or “BE” or “Esophagus adenocarcinoma” or “Esophagus cancer” were searched in GEO (www.ncbi.nlm.nih.gov/geo) database [8]. Then followed by the including criteria of selected datasets: (a) The used tissue should obtain from Barrett’s esophagus and Esophageal adenocarcinoma; (b) the microarray or RNA-sequencing data should include mRNA or miRNA; (c) at least 5 pair of samples were included.

The GSE16454 and GSE20099 miRNA expression profile data and three gene expression profiles (GSE13898, GSE26886, and GSE1420) were downloaded from the GEO database. The miRNA microarrays GSE16456 which was based on GPL16436 Human miRNA Microarray 1.0 platform was submitted by Yang et al. (2009), including 8 EAC and 10 BE [9]. The miRNA expression microarrays GSE20099 which was based on GPL8871OSU_CCC v4.0 platform was submitted by Fassan et al.(2010), including 11 EAC and 14 BE [10]. The mRNA expression microarrays GSE13898 which was based on GPL6102 Illumina human-6expressionbeadchip platform was submitted by Kim et al.(2011), including 64 EAC and 15 BE [11]. The mRNA expression microarrays GSE26886 which was based on GPL570Affymetrix Human Genome U133 Array platform was submitted by Wang et al.(2013), including 21 EAC and 20 BE [12]. The mRNA expression microarrays GSE1420 which was based on GPL96 Affymetrix Human Genome U133A Array platform was submitted by Khodarew et al.(2004), including 8 EAC and 8 BE [13]. For these datasets, only BE and EAC tissue samples were selected for further analysis.

### 2.2. miRNA and mRNA expression profiles

**Fig 1:**
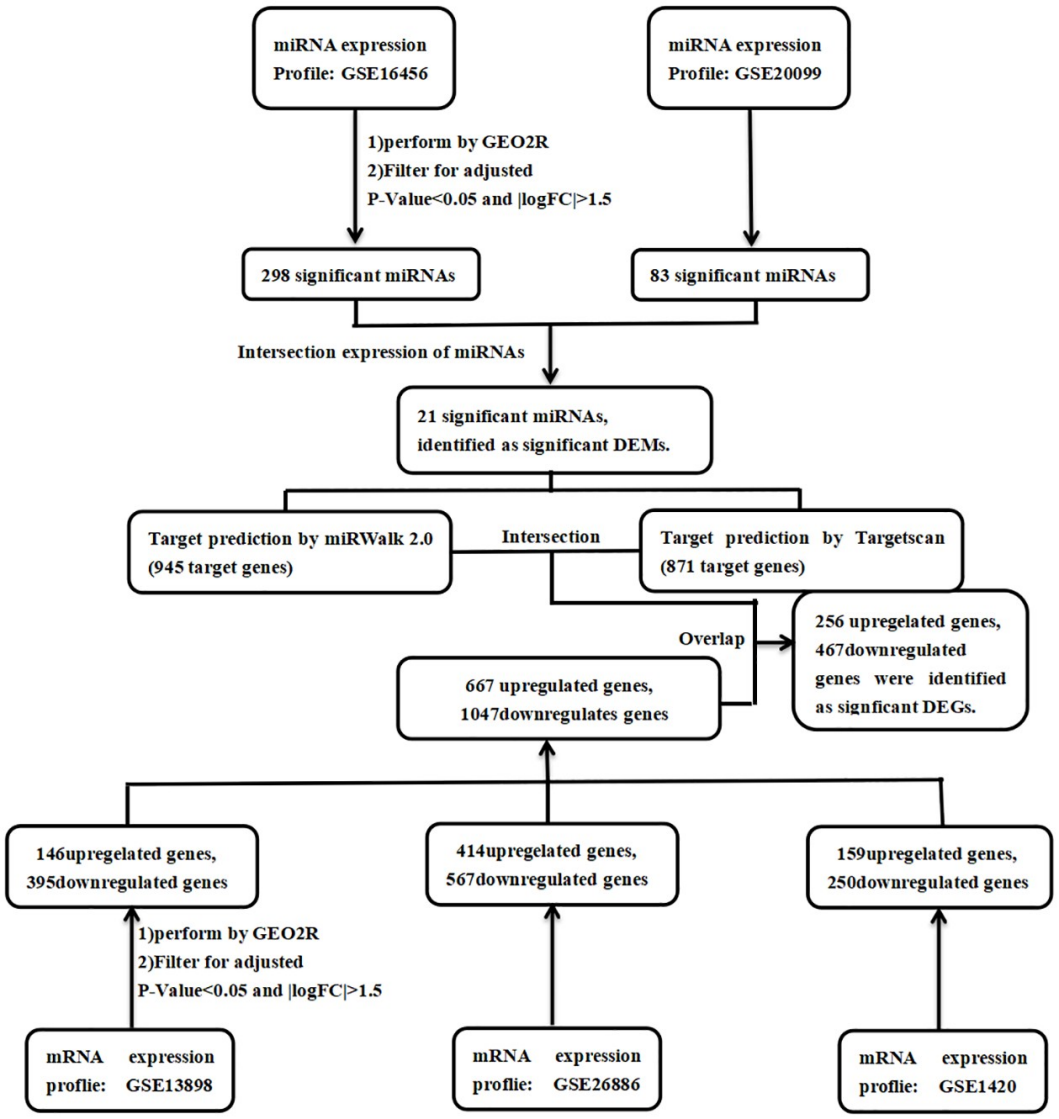
Flowchart of bioinformatics analysis.

The online analysis tool GEO2R (www.ncbi.nlm.nih.gov/geo/geo2r/) was used to screen the differentially expressed miRNAs (DEMs) and differentially expressed genes (DEGs) from the raw data. When the P value<0.05 and |logFC|≥1.5, the difference was regarded as statistically significant. The miRWalk 2.0 database (http://zmf.umm.uni-heidelberg.de/apps/zmf/mirwalk2/index.html) is a comprehensive and freely available database that provides a large number of predicted and experimentally verified miRNA-target interactions in a variety of novel ways, which provides great help for the study of miRNA [14]. Targetscan(http://www.targetscan.org/) [15] is a database for searching miRNA target genes of animals based on the evolutionary conservative characteristics of target gene sequences. We submitted the significant DEMs to Targetscan and miRWalk 2.0 database respectively to predict the target mRNAs. We selected the intersection of the target mRNAs predicted by the two databases, and then we extracted the significant DEGs by crossing the overlapping genes of target mRNA and significant DEMs (Fig 1).

### 2.3. Gene ontology and KEGG pathway analysis

The Database for Annotation, Visualization, and Integrated Discovery (DAVID, https://david-d.ncifcrf.gov/) provides a comprehensive set of functional annotation tools for investigators to understand biological meaning behind large list of genes [16]. Gene ontology (GO) and the Kyoto Encyclopedia of Genes and Genomes (KEGG) pathway enrichment analysis for DEGs used DAVID database. FDR <0.05 and gene count >2 were regarded as statistically significant [17].

### 2.4. Construction of the regulatory network

Search Tool for the Retrieval of Interacting Genes/Proteins (STRING, https://string-db.org/) is a database for online retireval of known protein-protein interactions (PPI) [18]. We submitted the DEGs to STRING data base and set the combined score >0.40 as the cut-off criteria which were based on experimental literature reports. Furthermore, we take the intersection of hub genes and significant DEGs (Intersection of predictions from miRWalk 2.0 and Targetscan). As the DEMs shared a common target mRNA with the hub genes of DEGs, we speculated they might exist in a similar regulatory pathway. Finally, we visualized the regulatory network describing miRNA and mRNA interaction using Cytoscape 3.7.0 [19].

### 2.5. Expression of significant DEGs and DEMs

The expression of significant DEGs was performed using GEPIA (http://gepia.cancer-pku.cn/index.html) [20]. The expression of significant DEMs was performed using UALCAN (http://ualcan.path.uab.edu/). Data analysis was performed using the TCGA database (https://cancergenome.nih.gov/) [21].

### 2.6. Survival analysis of DEGs and DEMs

A Kaplan–Meier analysis that based on data from the TCGA database (https://cancergenome.nih.gov/) was performed using Kaplan-Meier Plotter (https://kmplot.com/analysis/) [22]. In normal tissues, the expression levels of all genes were correlated with prognosis compared with EAC. There was a data included 184 EAC patients(157 males and 27 females) in the TCGA database. The P-value <0.05 was considered to be statistically significant. In view of the large differences in gender in the data set from TCGA, we combined the expression of mRNA or miRNA with gender to analyze the significant difference in survival rate.

## 3. Results

### 3.1. Identification of DEMs and DEGs

**Fig 2:**
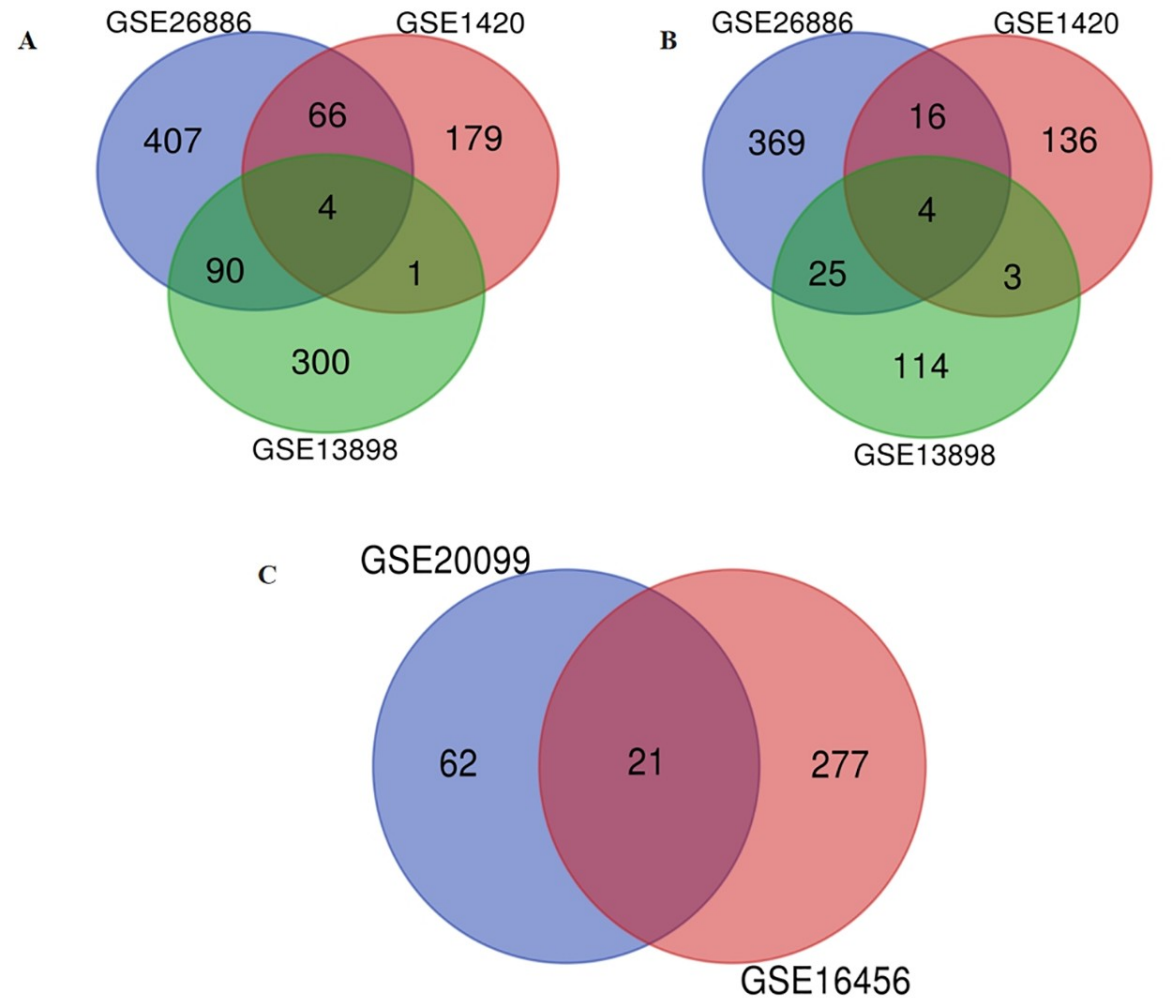
Identification of differentially expressed miRNAs (DEMs) and genes (DEGs). (A)Identification of downregulated DEGs; (B)Identification of upregulated DEGs; (C)Identification of DEMs.

A total of 21 DEMs were screened out from the GSE16456 and GSE20099 datasets as shown in Fig 2C. These significant miRNAs obtained have been listed in Table 1. Because miRNA may regulate mRNA in a positive or negative way, so we took the up-regulated and down-regulated DEMs together. As shown in Fig 2B and 2A, 667 up-regulated and 1047 down-regulated DEGs were found in EAC samples compared with BE samples. A total of 21 significant DEMs target genes of 12,413 and 13,693 were obtained from the miRWalk 2.0 database and Targetscan respectively. The intercrossed number of these candidate genes was 306 and 565 with up-regulated and down-regulated DEGs from miRWalk 2.0 database, and 360 and 585 with up-regulated and down-regulated DEGs from Targetscan respectively. Take the intersection of these candidate genes from miRWalk 2.0 database and Targetscan as the significant DEGs. Finally, 256 up-regulated and 467 down-regulated genes were regarded as the group of significant DEGs.

**Table 1:** The P value and |logFC|≥1.5 of significant DEMs.

DEMs	GSE16454		GSE26099	
	P value	|logFC|≥1.5	P value	|logFC|≥1.5
hsa-miR-520f	0.0384184	2.351701	9.41E-05	1.536217
hsa-miR-147	0.0479059	2.081228	1.33E-03	1.545722
hsa-miR-18b	0.0115893	2.182704	4.50E-04	1.506087
hsa-miR-518e	0.0218604	2.926247	2.27E-06	1.881674
hsa-miR-181d	0.0005164	1.584864	6.41E-03	1.598469
hsa-miR-214	0.029153	1.640488	1.58E-03	1.50794
hsa-miR-612	0.0391628	3.331992	4.23E-03	1.524565
hsa-miR-9*	0.0494411	2.768059	2.29E-03	1.536797
hsa-miR-496	0.0410621	3.287839	2.39E-07	1.713264
hsa-miR-133b	0.015027	2.586753	2.50E-04	1.906789
hsa-miR-143	0.0274212	1.995434	6.50E-03	1.576561
hsa-miR-185	0.0099928	1.557057	5.25E-04	1.509349
hsa-miR-20b	0.0184024	1.520991	4.76E-04	1.735738
hsa-miR-100	0.0041751	2.31764	4.30E-03	1.595422
hsa-miR-627	0.0369296	2.406204	8.29E-05	1.556626
hsa-miR-126*	0.0115968	2.586943	2.04E-03	1.537629
hsa-miR-145	0.0193225	2.043886	3.62E-02	1.54336
hsa-miR-517c	0.0453688	3.184864	1.70E-04	1.645947
hsa-miR-15b	0.0160865	1.537395	9.92E-05	1.715495
hsa-miR-635	0.0074216	4.902887	2.19E-02	1.57625
hsa-miR-605	0.0430913	1.593943	1.47E-03	1.697192

The * in hsa-miR-9* and hsa-miR-126* is part of the name of miRNAs, it has no statistical meaning.

### 3.2. Functional annotation analysis

GO ontology contains three categories: molecular cellular component (CC), biological process (BP) and function (MF). The most significant GO terms in MF ontology for up-regulated DEGs were the 3’-5’-exoribonuclease activity, ribonuclease activity, prenylated protein tyrosine phosphatase activity, signaling receptor binding, and protein binding, while for the down-regulated DEGs were alcohol dehydrogenase activity, cell adhesion molecule binding, cytoskeletal protein binding, oxidoreductase activity, alcohol dehydrogenase activity, zinc-dependent, and myosin V binding.

In CC ontology, the cell part was significantly enriched GO terms for up-regulated genes. In contrast, the GO terms of down-regulated genes were significantly enriched in membrane, plasma membrane, cell periphery, membrane part and plasma membrane region.

In BP ontology, the up-regulated genes were mainly enriched in positive regulation of the biological process, positive regulation of the cellular process, positive regulation of signal transduction, positive regulation of cell communication and positive regulation of signaling. The down-regulated genes were mainly enriched in regulation of biological quality, response to an organic substance, epithelial cell differentiation, regulation of protein localization and positive regulation of transport.

Six main KEGG pathways were represented in the up-regulated genes, including proteasome, RNA degradation, epstein-barr virus infection, glycosaminoglycan biosynthesis-chondroitin sulfate, osteoclast differentiation and kaposi’s sarcoma-associated herpesvirus infection; Downregulated genes included fatty acid degradation, oocyte meiosis, metabolic pathways, inflammatory mediator regulation of TRP channels, gastric acid secretion and amphetamine addiction which were presented in Table 2.

**Table 2:** Significantly enriched GO terms and KEGG pathways.

Category	Term	Description	Gene counts	FDR
**Upregulated**				
GO:0000175	MF	3’-5’-exoribonuclease activity	5	0.0045
GO:0004540	MF	Ribonuclease activity	6	0.0233
GO:0004727	MF	Prenylated protein tyrosine phosphatase activity	2	0.0341
GO:0005102	MF	Signaling receptor binding	27	0.0344
GO:0005515	MF	Protein binding	80	0.0454
GO:0044464	CC	Cell part	155	0.0297
GO:0048518	BP	Positive regulation of biological process	80	9.80E-05
GO:0048522	BP	Positive regulation of cellular process	74	9.80E-05
GO:0009967	BP	Positive regulation of signal transduction	34	0.0001
GO:0010647	BP	Positive regulation of cell communication	36	0.0001
GO:0023056	BP	Positive regulation of signaling	36	0.0001
hsa03050	KEGG	Proteasome	6	0.00083
hsa03018	KEGG	RNA degradation	7	0.0009
hsa05169	KEGG	Epstein-Barr virus infection	8	0.0278
hsa00532	KEGG	Glycosaminoglycan biosynthesis—chondroitin sulfate	3	0.0481
hsa04380	KEGG	Osteoclast differentiation	6	0.0481
hsa05167	KEGG	Herpesvirus infection	7	0.0481
**Downregulated**				
GO:0004022	MF	Alcohol dehydrogenase (NAD) activity	4	0.0189
GO:0050839	MF	Cell adhesion molecule binding	12	0.019
GO:0008092	MF	Cytoskeletal protein binding	28	0.0203
GO:0016616	MF	Oxidoreductase activity	9	0.0203
GO:0004024	MF	Alcohol dehydrogenase activity, zinc-dependent	3	0.0289
GO:0031489	MF	Myosin V binding	4	0.0289
GO:0016020	CC	Membrane	165	4.28E-05
GO:0005886	CC	Plasma membrane	112	0.00015
GO:0071944	CC	Cell periphery	113	0.00015
GO:0044425	CC	Membrane part	132	0.00018
GO:0098590	CC	Plasma membrane region	36	0.00023
GO:0065008	BP	Regulation of biological quality	94	3.35E-06
GO:0010033	BP	Response to organic substance	77	2.62E-05
GO:0030855	BP	Epithelial cell differentiation	29	0.00017
GO:0032880	BP	Regulation of protein localization	34	0.00046
GO:0051050	BP	Positive regulation of transport	33	0.00084
hsa00071	KEGG	Fatty acid degradation	6	0.0173
hsa04114	KEGG	Oocyte meiosis	9	0.0173
hsa01100	KEGG	Metabolic pathways	33	0.0406
hsa04750	KEGG	Inflammatory mediator regulation of TRP channels	7	0.0406
hsa04971	KEGG	Gastric acid secretion	6	0.0406
hsa05031	KEGG	Amphetamine addiction	6	0.0406

BP = biological process, CC = cellular component, FDR = false discovery rate, GO = gene ontology, KEGG = Kyoto Encyclopedia of Genes and Genomes, MF = molecular function

### 3.3. PPI network

**Fig 3:**
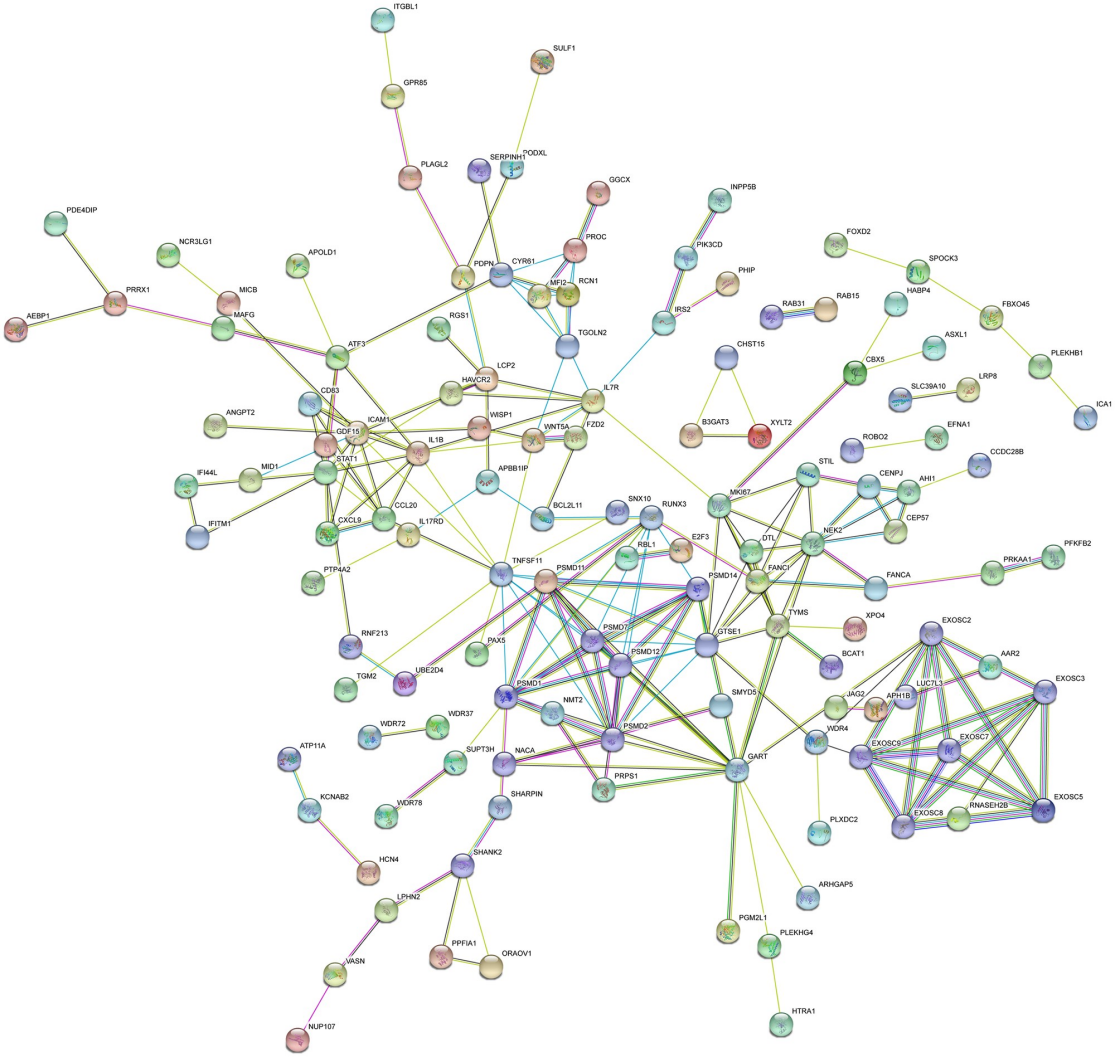
PPI networks of significantly upregulated DEGs.

**Fig 4:**
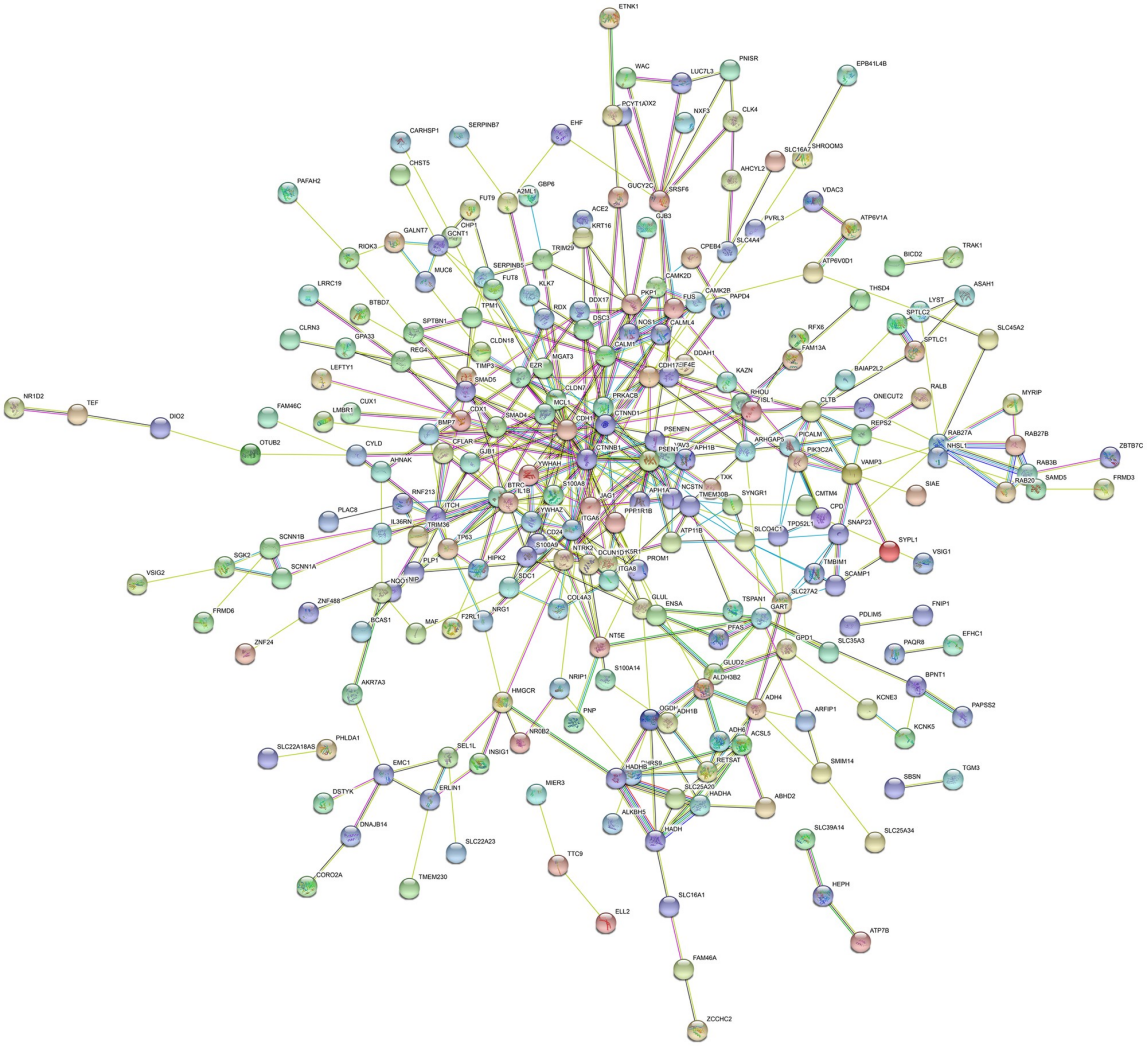
PPI networks of significantly downregulated DEGs.

The PPI network of DEGs was based on STRING. A total of 176 nodes and 220 edges were mapped in the PPI network of significantly up-regulated DEGs (Fig 3). While, 290 nodes and 463 edges constituted the network of significantly down-regulated DEGs (Fig 4). In PPI network, the edge was essential when detecting the hub genes. The parameter “degree” was used to calculate the edge count of each gene in PPI network. Table 3 showed the top 5% degree genes evaluated as hub genes. Sixteen genes were selected from PPI network as hub genes of EAC. These hub genes might play a key role in EAC.

**Table 3:** Top 5% hub genes in the PPI networks.

Ensenmbl gene ID	Gene symbol	Full gene name	degree
**Upregulated**			
ENSG00000159131	GART	Phosphoribosylglycinamide formyltransferase	14
ENSG00000120659	TNFSF11	TNF superfamily member 11	13
ENSG00000075218	GTSE1	G2 and S-phase expressed 1	12
ENSG00000117650	NEK2	NIMA related kinase 2	11
ENSG00000090339	ICAM1	Intercellular adhesion molecule 1	11
ENSG00000197170	PSMD12	Proteasome 26S subunit, non-ATPase 12	10
**Downregulated**			
ENSG00000168036	CTNNB1	Catenin beta 1	32
ENSG00000039068	CDH1	Cadherin 1	28
ENSG00000080815	PSEN1	Presenilin 1	18
ENSG00000125538	IL1B	Interleukin 1 beta	16
ENSG00000198561	CTNND1	Catenin delta 1	14
ENSG00000101384	JAG1	Jagged canonical Notch ligand 1	14
ENSG00000079112	CDH17	Cadherin 17	13
ENSG00000078747	ITCH	Itchy E3 ubiquitin protein ligase	13
ENSG00000198668	CALM1	Calmodulin 1	12
ENSG00000091409	ITGA6	Integrin subunit alpha 6	12

Colored nodes: query proteins and first shell of interactors; white nodes: second shell of interactors; Blue-green line: known interactions from curated databases; purple line: known interactions from experimentally determined; green line: predicted interactions from gene neighborhood; red line: predicted interactions from gene fusions; dark blue: predicted interactions from gene co-occurrence; yellow line: interactions from textmining; black line: interactions from co-expression; light blue: interactions from protein homology.

Colored nodes: query proteins and first shell of interactors; white nodes: second shell of interactors; Blue-green line: known interactions from curated databases; purple line: known interactions from experimentally determined; green line: predicted interactions from gene neighborhood; red line: predicted interactions from gene fusions; dark blue: predicted interactions from gene co-occurrence; yellow line: interactions from textmining; black line: interactions from co-expression; light blue: interactions from protein homology.

### 3.4. miRNA–mRNA interaction network

**Fig 5:**
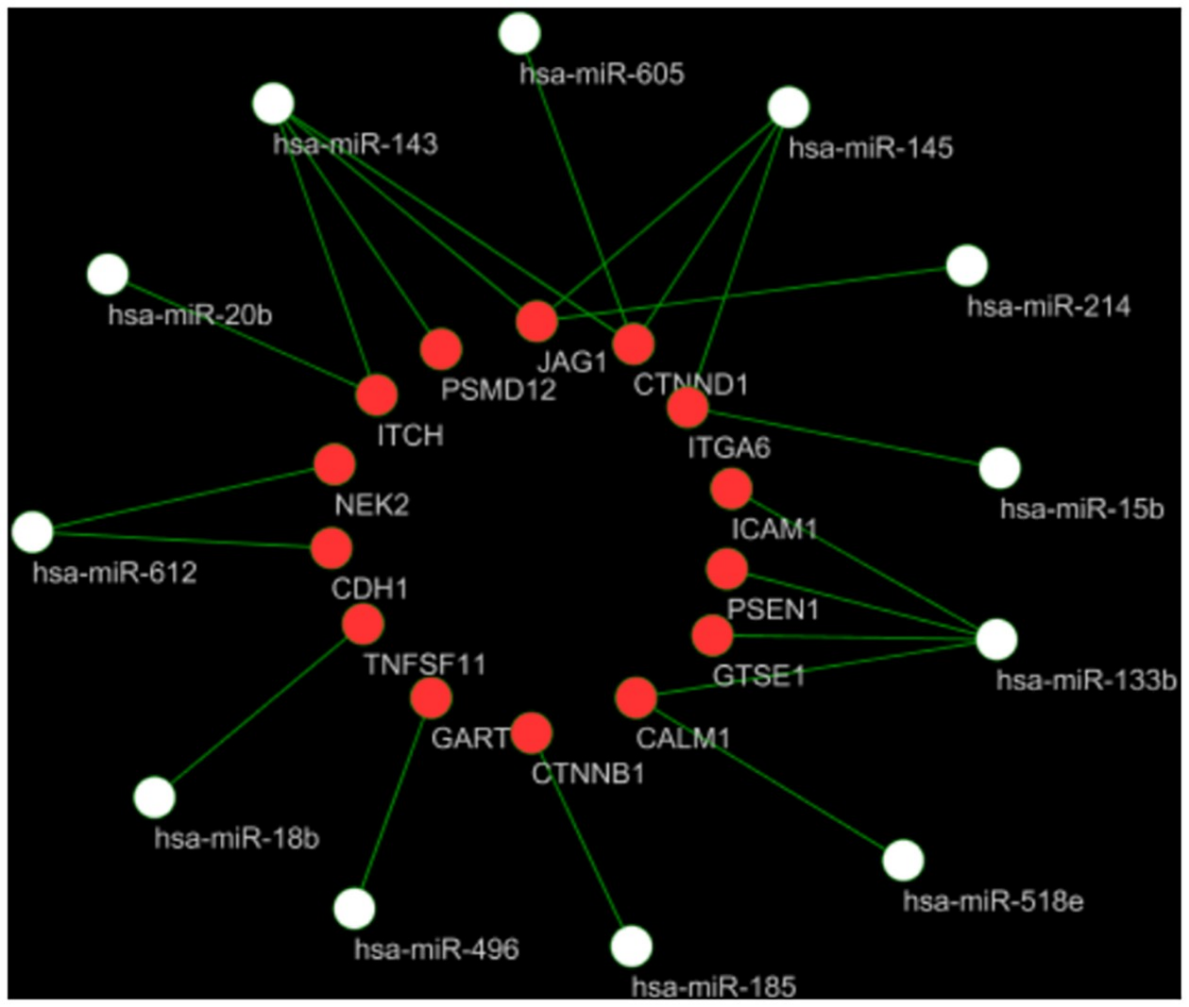
The miRNA-mRNA regulatory network. White nodes, miRNA; Red nodes, mRNA.

In order to further investigate the mutual regulatory relationship among 21 significant DEMs and hub genes, we built the miRNA-mRNA regulatory network (Fig 5). On the one hand, hsa-miR-143 and hsa-miR-133b was the highest connectivity target genes. On the other hand, some of the hub genes were calculated to be common targets for different miRNAs. For example, JAG1 might be the common target of hsa-miR-214, hsa-miR-143 and hsa-miR-145. No hub genes could be used as a target gene for hsa-miR-520f, hsa-miR-147, hsa-miR-181d, hsa-miR-9*, hsa-miR-627, hsa-miR-126, hsa-miR-635 and hsa-miR-517c.

### 3.5. Expression of significant DEGs and DEMs

**Fig 6:**
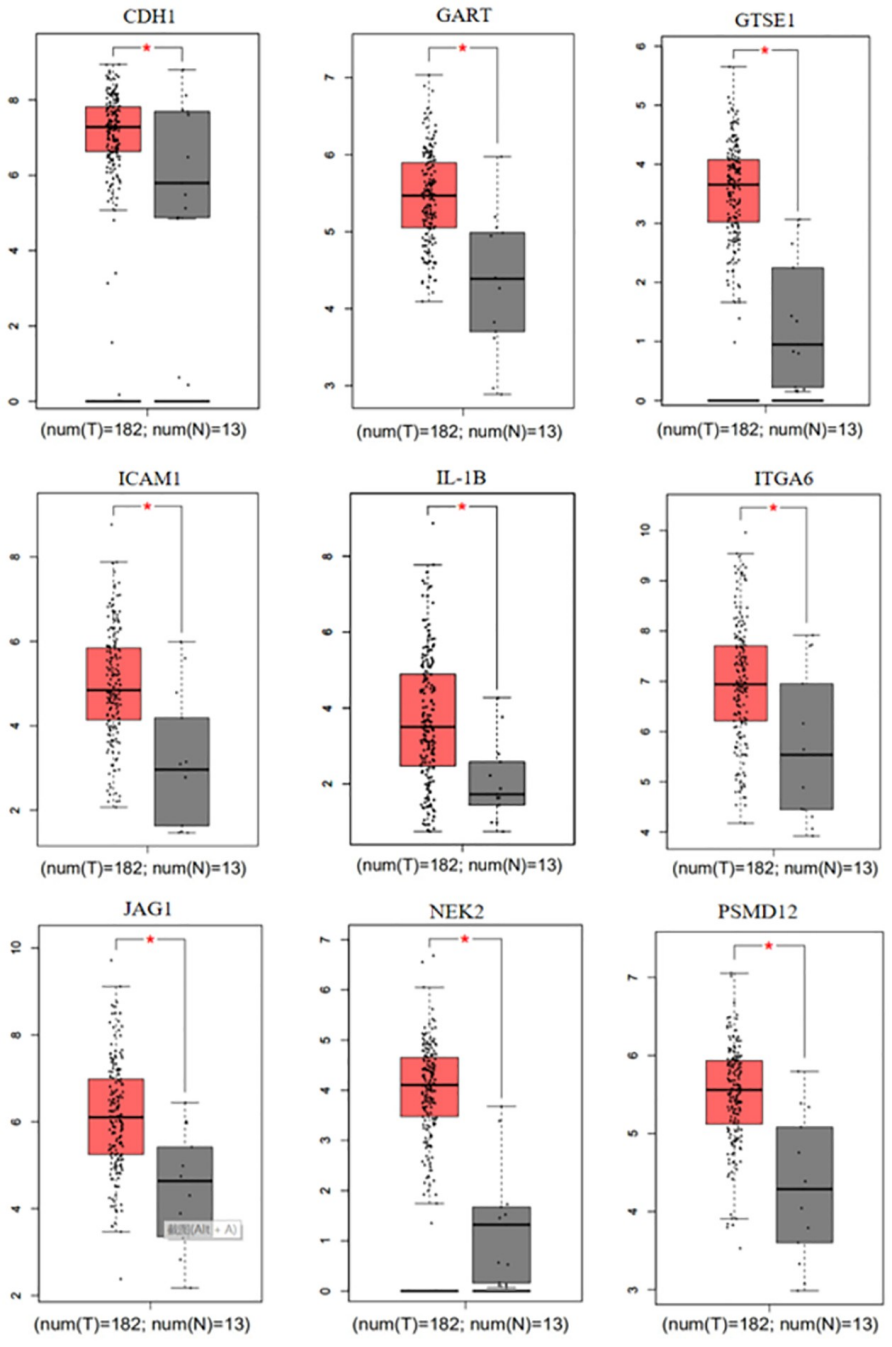
Expression of significant DEGs in the TCGA dataset. Expression of significant DEGs between EAC and normal tissue in the TCGA dataset. Red column represents the expression of EAC, gray column represents the expression of normal tissue. * respresents the P value <0.05.

**Fig 7:**
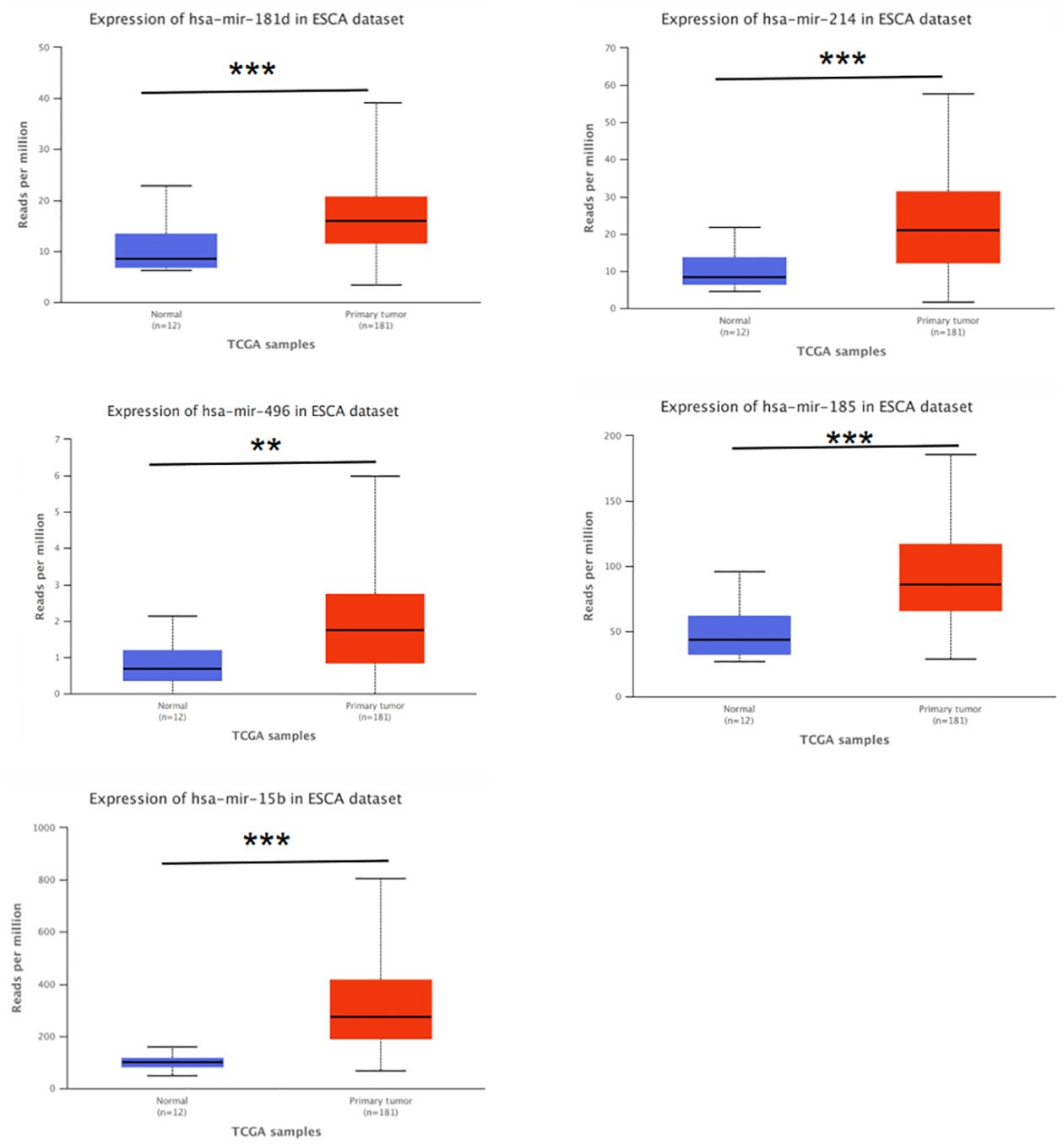
Expression of significant DEMs in the TCGA datasets. Expression of significant DEMs between EAC and normal tissue in the TCGA dataset. Red column represents the expression of EAC, blue column represents the expression of normal tissue. The horizontal axis represents different specimens, the vertical axis represents the number of reads from a gene per kilobase length per million reads. * respresents the P value <0.05, ** respresents the P value <0.01, *** respresents the P value <0.001.

**Fig 8:**
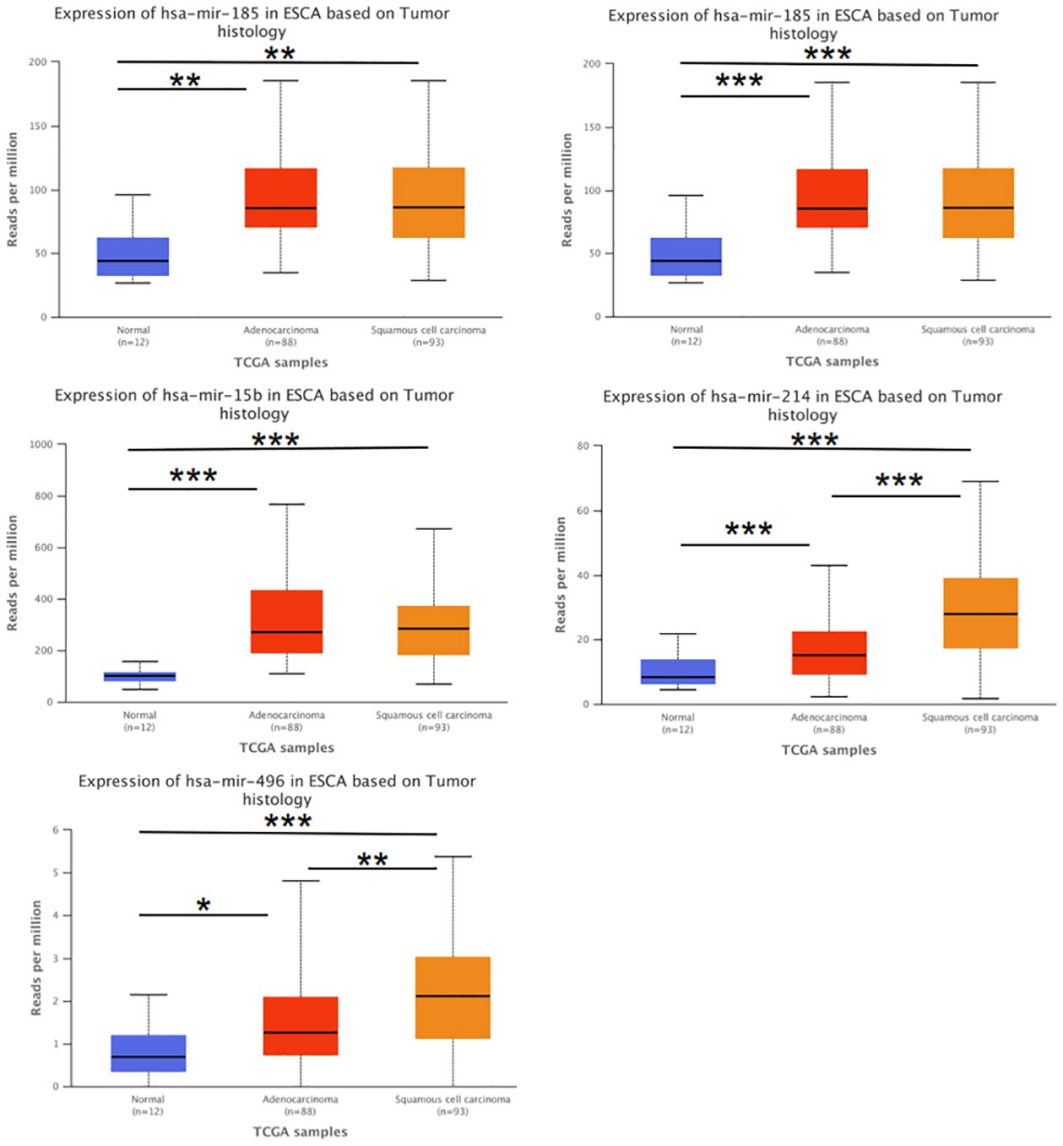
Expression of significant DEMs in the TCGA dataset between normal tissue, EAC and ESCC. Expression of significant DEMs between EAC, ESCC and normal tissue in the TCGA dataset. Blue column represents the expression of normal tissue, red column represents the expression of EAC and yellow column represents the expression of ESCC. The horizontal axis represents different specimens, and the vertical axis represents the number of reads from a gene per kilobase length per million reads. * respresents the P value <0.05, ** respresents the P value <0.01, *** respresents the P value <0.001.

We investigated the DEGs expression level in the TCGA dataset and found that 9 mRNAs in EAC were significantly different from normal tissue (Fig 6). We examined the 21 significant DEMs in the TCGA dataset, and compared their expression trends with the GEO databases. Of which, 17 miRNAs were consistent between the two databases. Five of the 17 miRNAs were significantly different between the normal tissue and the ESCA tissue (Fig 7). Next, we explored the different expressions of the five miRNAs in normal tissue, EAC and ESCC (Fig 8). The expression level of hsa-miR-181d, hsa-miR-185 and hsa-miR-15b were remarkable different between normal tissue and EAC, normal tissue and ESCC, but not between ESCC and EAC. Moreover, there was obvious difference in the expression of hsa-miR-214 and hsa-miR-496 between normal tissue, EAC, and ESCC. However, the expression trends of hsa-miR-520f, hsa-miR-9*, hsa-miR-517c, and hsa-miR-627 were not consistent with the GEO data.

### 3.6. Survival analysis of miRNA/mRNA in EAC

**Fig 9:**
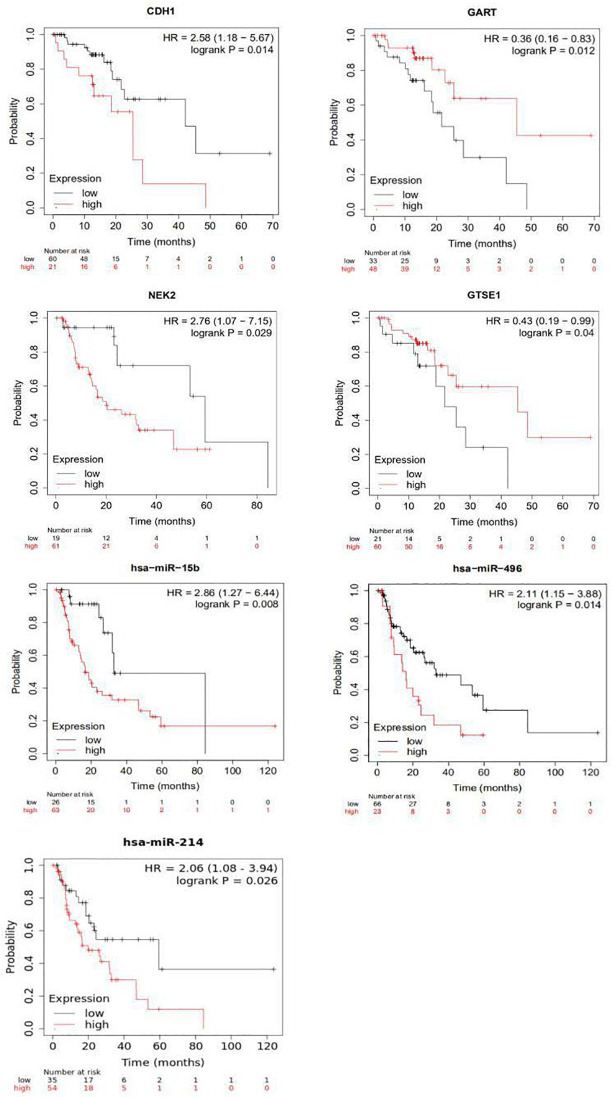
Prognostic values of DEMs and DEGs for overall survival in EAC patients. EAC patients were divided into low and high expression groups. Red polylines and text represent high expression groups, and gray polylines and text represent low expression groups. N represents the number of patients in each group. The horizontal axis represents the survival time in months, and the vertical axis represents the survival rate of patients in the corresponding time. Number at risk represents the number of patients who survived at the corresponding time point.

**Fig 10:**
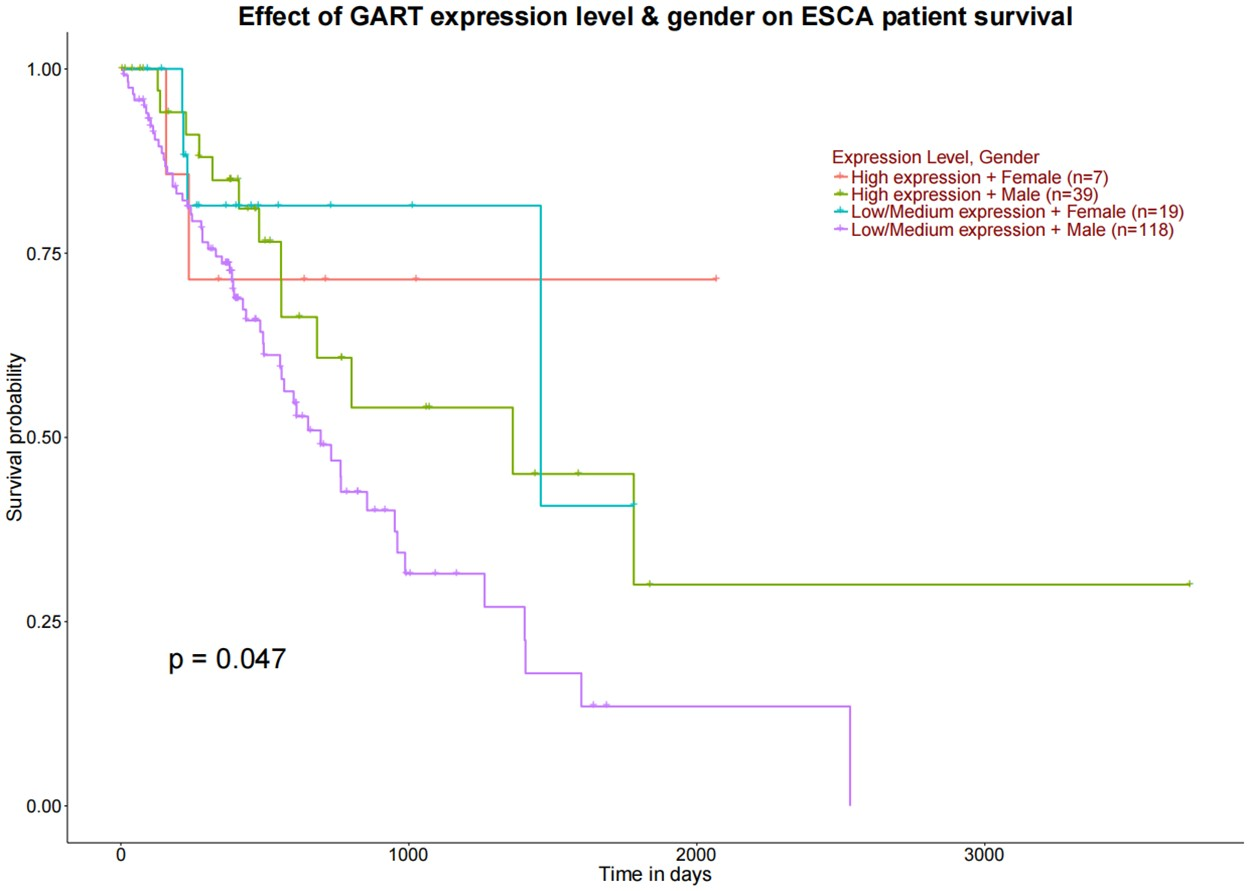
Combined the expression of mRNA or miRNA with gender, GART was correlated to overall survival (OS) in EAC patients.

Based on the TCGA, survival analysis was conducted among the 9 mRNAs and 5 miRNAs, as mentioned above. Results from the Kaplan-Meier method [23] and the log-rank test showed that CDH1, GART, GTSE1, NEK2 and hsa-miR-496, hsa-miR-214, hsa-miR-15b were correlated to overall survival (OS) in EAC patients (Fig 9 and Table 4). When combined the expression of mRNA or miRNA with gender, only GART was correlated to overall survival (OS) in EAC patients (Fig 10).

**Table 4:** The significant DEMs and DEGs related to overall survival.

Gene symbol	High expression	*P*	HR
DEGs			
GART	Tumor	0.012	0.36
CDH1	Tumor	0.014	2.58
NEK2	Tumor	0.029	2.76
GTSE1	Tumor	0.04	0.43
DEMs			
hsa-miR-15b	Tumor	0.008	2.86
hsa-miR-496	Tumor	0.014	2.11
hsa-miR-214	Tumor	0.026	2.06

## 4. Discussion

Globally, squamous cell carcinoma is the most common type that accounts for the vast majority of EC cases. Yet, over recent years, the proportion of EAC has been dramatically increasing in affluent nations, including China [24]. It is believed that most of EAC develop from BE that is a long-term and poorly understood process. Once the dysplasia breaks through the basement membrane, tumor cells infiltrate, and the disease rapidly progresses. The 5-year survival rate of patients with EAC is less than 20% [25].

Despite great progress in diagnosis, the molecular mechanisms involved in the BE progressing into EAC have not been clarified [26]. Therefore, to identifying the molecular targets for diagnosis and treatment have become of essential and urgent importance. In this study, we found that the DEGs were mainly concentrated in specific pathways, including Epstein-Barr virus infection, herpesvirus infection, fatty acid degradation, gastric acid secretion and TRP channels. The relationship between pathogen infection and tumorigenesis has always been a focus of interest in oncology. It is estimated that more than 200,000 cancer patients and 2% of cancer-related deaths worldwide are associated with viral infection each year [27]. The main virus that can directly affect the formation of a malignant epithelial tumor is Epstein Barr virus (EBV) and human papillomavirus (HPV) [28]. HPV infection has been strongly associated with the occurrence of urogenital tumor, such as cervical cancer, the cancer of the penis, oral cancer as well as anal cancer [29], while EBV infection is closely relatedwith digestive tract related tumors, nasopharyngeal carcinoma, leiomyosarcoma, Burkitt lymphoma, Hodgkin’s and non-Hodgkin’s lymphoma [23, 30]. HPV is a virus with double stranded DNA structure. It is found that HPV can integrate into the host genome, induce DNA damage by changing cell cycle and telomere protein, block tumor suppressor related signal pathway and apoptosis process, lead to tissue malignant transformation and eventually develop into cancer [31]. Moreover, a general early integration between the virus and the host gene was found in patient infected with HPV, the integration degree was significantly related to the severity of the disease [32]. It is speculated that the micro-environment of HPV persistent infection caused by the integration of the HPV genome with the host chromosome is one of the key factors for BE progression to EAC [33]. Yet, the connection between EBV infection and the occurrence of esophageal cancer still remains debatable. Previous studies have suggested that EBV may appear through tumor-infiltrating lymphocytes in some advanced lesions. Latest research show that EBV infection was significantly correlated with ARID1A and PD-L1 expressions and CD8^+^ TILs in GCs [34]. Infection with EBV can induce the hypermethylation of both host and viral genomes, which regulate cellular functions to facilitate immune evasion and viral persistence. So, newest view divides the EBV-associated gastric cancer (EBVaGC) into a distinct subtype of gastric cancer [35]. Clinically, EBVaGC has a lower frequency of lymph node metastasis and better prognosis than EBV negative gastric cancer. Moreover, EBV infection has been correlated with gender, lymph node metastasis and tumor location in patients with gastrointestinal cancer [36]. The research on Epstein Barr virus and lymphoma is also controversial. The traditional view is that EB virus is one of the pathogenic factors of lymphoma and belongs to “first hit”. The latest research suggests that EB virus infection is a secondary event of lymphoma, not the first. According to the above-mentioned EB virus patients have a lower risk of lymphatic metastasis, the relationship between EB virus infection and lymphatic circulation still needs to be further studied [37].

We found that CDH1, GART, GTSE1, NEK2, and hsa-miR-496, hsa-miR-214, and hsa-miR-15b were proved to be associated with survival, which indicates that they might not only regulate the cellular process but could also have important clinical application value. GERD, which is induced by a disorder of fatty acid metabolism and an increase of gastric acid secretion, was considered to be the most important risk factor for the progression of BE to EAC. On the one hand, the long-term, repeated chronic inflammation induced by gastric acid and fatty acid form can lead to serious DNA damage (base mismatch). On the other hand, the inflammatory microenvironment inhibits DNA repair in GERD patients [38], which was the direct cause leading to BE and EAC.

The TRP channel transduction pathway is closely related to the taste and pain of the digestive system [39]. The abnormal expression of the TRP channel in esophageal carcinoma can promote the proliferation, migration, invasion and differentiation of cancer cells. TRPC1, a vital node molecule in the TRP channel, is related to the stage of EC [40]. It can also be used as a predictor of the survival time of SC patients. TRPC6 mRNA expression levels are increased in human EC tissues compared to normal tissues [40]. The knock-down and inhibition of TRPM8 may decrease the proliferation of EC cells [41]. In addition, a higher expression of TRPV2 protein has been shown to be correlated with a worse 5-year overall survival rate after surgery [42].

Increasing evidence has suggested that the deep involvement of miRNAs can function as tumor suppressors or oncogenes in carcinogenesis. Several studies have focused on miRNAs’ significance in BE and EAC, revealing the potential of miRNA profiles for distinguishing BE tissue from EAC and identifying BE patients at high risk of progression to EAC [43–45]. However, they did not deeply report on the effect of the miRNA-mRNA networks. Hence, the identification of the miRNA-mRNA regulatory network is of great significance to the further study of EAC. Compared with normal samples, 21 significant DEMs were identified. Among them, hsa-miR-147e [46], hsa-miR-181d [47], hsa-miR-214 [48, 49], hsa-miR-612 [50], hsa-miR-133b [51], hsa-miR-143 [52–55], hsa-miR-100 [56], hsa-miR-126* [57], hsa-miR-145 [52, 58–60], hsa-miR-15b [61] were all reported in EC. Most importantly, hsa-miR-496, hsa-miR-214, hsa-miR-15b were found to be correlated with patient survival. Hsa-miR-214 has been strongly associated with carcinogenesis. Previous studies reported that miR-214 targets LZTS1 through PI3K/AKT/mTOR signaling pathway, promotes ESCC cells proliferation, migration, invasion and inhibits apoptosis [49]. In breast cancer cells, depletion of miR-214 can inhibit the vascular endothelial pathway of malignant cells by reducing the expression of the cell adhesion molecules ITGA5 and ALCAM [62]. In colon cancer, miR-214 targeting BCL9L can inhibit proliferation, metastasis, and epithelial-mesenchymal transition by down-regulating Wnt signaling [63]. Moreover, miR-214 has also been associated with osteoporosis, osteosarcoma, multiple myeloma, and osteolytic bone metastasis of cancer [64].

Brain-derived neurotrophic factor (BDNF) was suggested as a potential target material of miR-496 [65]. Inactivating BDNF-mediated PI3K/Akt signaling pathway activation could increase expression of miR-496 which was regarded as suppress tumor growth [65]. Another research proved that miR-496 could regulate mTOR expression by directly binding to LnvRNA-DANCR in lung adenocarcinoma [66]. LncRNA-HCG11 can interact with the miR-496/CPEB3 axis to inhibit glioma progression [67].

Hsa-miR-15b can be used as a biomarker to discriminate human ovarian cancer tissues from normal tissues. The sensitivity and specificity of it were 97% and 92% respectively [68]. The overexpression of hsa-miR-15 can promote cisplatin resistance of lung adenocarcinoma cells by inhibiting the expression of phosphatidylethanolamine binding protein 4 (PEBP4) [69]. Through bioinformatic methods, hsa-miR-15b was forecasted to contribute to the pathogenesis of non-small cell lung cancer [70], breast cancer [71]https://pubmed.ncbi.nlm.nih.gov/20301167/, gastric cancer [72] and colorectal cancer [73]. In conclusion, these important DEMs offered potential biomarkers and molecular mechanisms for the high-risk diagnosis of BE.

The overall changes of mRNA and miRNA expression are associated with the regulatory mechanisms of the development and progression of BE. 16 mRNAs has been identified, which were seem as hub genes, might have crucial roles in EAC. CDH1, GART, GTSE1 and NEK2 were found to be correlated with survival. CDH1, which is considered to be the driving gene of BE progressing to EAC, is strongly expressed in the BE [62]. CDH1 is mainly localized on the plasma membrane and functions as a gatekeeper of the epithelial cell. The expression of CDH1 in BE without dysplasia was similar to that in the squamous epithelium. Yet, the expression of CDH1 significantly changed during the progression of BE to EAC. In poorly differentiated EAC, the expression level was almost zero. This phenomenon suggests that low expression of CDH1 might be a marker of high-risk transformation from BE to EAC. Moreover, patients with CDH1 mutations are more at risk of diffuse gastric cancer and lobular breast cancer [74]. It has been reported that the cumulative risk of diffuse gastric cancer at age of 80 years is 70% for men CDH1 mutation carriers and 56% for women [75].

GART has been shown to be related to digestive cancer by mediating a metastatic cascade [76]. Elevated expression of GART, which is associated with chemosensitivity to multiple drugs, has been used as a target for anti-cancer drugs [77–79]. The depletion of GART can inhibit cell proliferation and blocked mitosis. In addition, GART can indicate poor prognosis in liver cancer. GTSE1 could promote the growth of cancer cell via activating the AKT pathway and promote tumor metastasis by EMT pathway [80]. The overexpression of GTSE1 might be involves in regulating FoxM1/CCNB1 expression by inducting lymph node invasion and progression. Patients with higher expression of GTSE1 were more likely to have a shorter survival time [3].

NEK2 is highly expressed in various tumor types and cancer cell lines with rapid relapse and poor outcome [81, 82]. Studies have found that overexpression of NEK2 may lead to chromosomal instability, mitosis, and aneuploidy, which is associated with the invasion, metastasis, proliferation, apoptosis, and sensitivity of a variety of tumors [82]. These processes include PP1/AKT, WNT signaling pathway and Ki-67. Inhibition of NEK2 expression can significantly inhibit tumor growth in vivo and in vitro [82], and NEK2 was also identified as a hub gene in ESCC [83]. Therefore, we speculate that NEK2 may become the next therapeutic target of EC.

## 5. Conclusion

In this research, 21 DEMs and 723 DEGs (256 up-regulated and 467 down-regulated) were identified. CDH1, GART, GTSE1, NEK2 and hsa-miR-496, hsa-miR-214, hsa-miR-15b were found to be correlated with survival and may be potential molecular biomarkers for predicting the clinical risk of BE patient progressing to EAC.

## Abbreviations

BE: Barrett’s esophagus
BP: biological process
CC: cellular component
CDH1: cadherin 1
DAVID: database for annotation, visualization and integrated discovery
DEGs: differentially expressed genes
DEMs: differentially expressed miRNAs
EAC: esophageal adenocarcinoma
EC: esophageal carcinoma
FDR: false discovery rate
GART: phosphoribosylglycinamide formyltransferase
GEO: gene expression omnibus
GO: gene ontology
GTSE1: G2 and S-phase expressed 1
KEGG: Kyoto Encyclopedia of Genes and Genomes
MF: molecular function
NEK2: NIMA related kinase 2
OS: overall survival
PPI: protein–protein interactions
STRING: search tool for the retrieval of interacting genes/proteins
